# Assessment of efficacy and tolerability of once-daily extended release metformin in patients with type 2 diabetes mellitus

**DOI:** 10.1186/1758-5996-2-16

**Published:** 2010-03-18

**Authors:** Juliana Levy, Roberta A Cobas, Marília B Gomes

**Affiliations:** 1 Department of Medicine, Diabetes Unit, State University of Rio de Janeiro, Rio de Janeiro, Brazil

## Abstract

**Aims:**

To determine prospectively the efficacy, tolerability and patient satisfaction of an extended release formulation of metformin (metformin XR) in hospital based outpatients with type 2 diabetes mellitus currently treated with standard metformin.

**Methods:**

Patients on immediate release standard metformin either alone or combined with other oral agents were switched to extended release metformin XR 500 mg tablets and titrated to a maximum dose of 2000 mg/day Measurements to include glucose and lipid control, blood pressure, body weight, waist circumference, C-reactive protein, adverse events and patient satisfaction were recorded at baseline, three and six months.

**Results:**

Complete data were obtained for 35 of the 61 patients enrolled to the study. At three and six months no changes were reported for any of the cardiovascular risk factors except for lipids where there was a modest rise in plasma triglycerides. These effects were achieved with a reduced dose of metformin XR compared to pre-study dosing with standard metformin (1500 mg +/- 402 vs 1861 +/- 711 p = 0.004). A total of 77% of patients were free of gastrointestinal side effects and 83% of patients stated a preference for metformin XR at the end of the study. Ghost tablets were reported in the faeces by the majority of the patients (54.1%).

**Conclusions:**

Patients switched to extended release metformin XR derived the same clinical and metabolic benefits as for standard metformin but with reduced dosage, fewer gastrointestinal side effects and a greater sense of well being and satisfaction on medication.

## Introduction

Type 2 Diabetes Mellitus is a chronic and progressive disease which demands intense management from diagnosis and through the various stages of the disease [1,2]. Its epidemic proportions worldwide [3], including Brazil [4], make it a public health issue and the present goal is to implement policies aimed at both its prevention [5,6] and management [7,8].

Lifestyle change and the use of pharmaceutical therapies are considered pivotal to achieving good control of glycaemia and other cardiovascular risk factors in the expectation of avoiding long-term complications and premature mortality [9]. Metformin is recommended in International Guidelines [7,8] as first line therapy due to its favourable profile on metabolic indices of glucose, lipid and weight control [10] as well as offering protection from life threatening complications and premature mortality [11,12].

Standard metformin suffers from the limitations of having to be administered two or three times a day and with the attendant risk of triggering gastrointestinal symptoms affecting 25% of patients making dose optimization problematic [13,14]. The joint consensus statement from the American Diabetes Association (ADA) and the European Society for the study of Diabetes (EASD) recognize these difficulties and give advice on how to minimize poor compliance with standard metformin [8]. Compliance is an issue for all chronic diseases [15] and poor tablet adherence is a particular concern in managing patients with diabetes due to the burden of medication [16,17]. In the 5 point plan for introducing metformin, the ADA/EASD draw attention to the recently introduced extended release metformin. This prospective study sets out to determine the ability of hospital based outpatients with type 2 diabetes to switch from standard metformin to extended release formulation (metformin XR). Moreover, to assess the resultant benefits on cardiovascular risk factors including glycaemia, plasma lipids, blood pressure, body weight and anthropometric measurements as well as measures of tolerability and patient satisfaction.

## Patients and Methods

### Study design

This was an open-label, prospective 24-week study conducted in patients with type 2 diabetes who were outpatients of the diabetes clinic at the State University of Rio de Janeiro. Approved by the local ethics committee, patient enrollment was restricted to the following patients: those aged 18 years and greater and taking immediate release metformin alone or in combination with other oral agents, had symptoms of poor diabetes control, were pregnant, serum creatinine levels > 1.3 mg/dL (female) or 1.4 mg/dL (male), nephropathy, evidence of hepatic disease or history of alcohol abuse.

Assessments were at baseline whilst on immediate release metformin, then at 3 and 6 months respectively following switchover to the once-a-day extended release metformin (Diabex XR or Glucophage SR/XR in Europe). At baseline, patients were started on the 500 mg XR tablet taken with the evening meal and titrated up to a maximum of four tablets per day.

The effectiveness of the extended release tablet was measured on three cardiovascular risk factors - blood glucose [HbA1c, fasting blood glucose (FBG), and postprandial blood glucose (PPBG)] blood lipids [total cholesterol, LDL cholesterol (Friedwald calculation) HDL cholesterol and triglycerides] and blood pressure. Body weight was measured as BMI, as were the anthropometric measures of waist circumference and waist/hip ratio (WHR). Other laboratory measurements included serum high sensitive C-reactive protein (CRP) the albumin excretion rate (AER) and vitamin B12 levels.

Gastrointestinal tolerability and adverse events were assessed at baseline and on completion of the study. All reported events were classified as unrelated or related to the study medication. Patient satisfaction forms were completed by patients to gain a measure of acceptability of the extended release tablet over standard metformin.

## Methods

All patients passed urine immediately before 8 p.m., discarded this sample and recorded the time. All the urine passed until 6 a.m. was collected into containers without a preservative. The urine volume was recorded, and aliquots were stored in glass tubes at - 70° Celsius until analysis. Fasting blood samples were also obtained. The urinary albumin concentration was estimated by solid-phase competitive chemiluminescent enzyme immunoassay (Immulite 1000 Systems, DPC Medlab, Los Angeles, CA, sensitivity of 0.5 ug/ml) with intra-assay and inter-assay coefficients of variation of 4.4% and 6.1%, respectively. Based on the AER in at least two out of three overnight urine specimens, patients were divided into two groups: Microalbuminuria (AER ≥ 20 ug/min and < 200 ug/min in two out of three overnight urine specimens) and normoalbuminuria (AER < 20 ug/min in two out of three overnight urine specimens). Each urine specimen was tested for bacteriuria, and when the latter was present (> 10^5^/mm^3^), it was discarded.

Blood pressure was measured by the same observer 3 times after a 5-min rest in the supine position using a standard mercury sphygmomanometer. Diastolic blood pressure (dBP) was recorded at the disappearance of Korotkoff sounds (phase 5). The mean of the measurements of systolic and diastolic blood pressure was used. Hypertension was defined as systolic blood pressure (sBP) > 140 mmHg and/or dBP > 90 mm Hg [2] or any value in patients under antihypertensive treatment. Blood samples were drawn in the morning between 7:30 and 8:30 a.m., after the last urine collection and an overnight fast. After centrifugation at 2500 × g for 15 min at room temperature (19°Celsius), aliquots of plasma and sera were stored at -70° Celsius until analysis. Serum CRP (mg/dl) was measured using a highly sensitive immunonephelometry assay (Behring Nephelometer, Germany) with a detection limit of 0.01 mg/dl and with intra-assay and inter-assay CV of 1% and 5.3%, respectively. Vitamin B12 was determined by an enzyme immunoassay method (reference value: 180-914 pg/mL).

Creatinine (mg/dl), FBG (mg/dl), postprandial plasma glucose (PPG, mg/dl), triglyceride (mg/dl), HDL cholesterol (mg/dl) and total cholesterol (mg/dl) levels were measured using an auto-analyzer (Cobas-Mira Roche) by enzymatic techniques. Postprandial glucose was measured two hours after the usual breakfast. Patients were instructed to take their usual medication.

Body mass index (BMI) was calculated by dividing the weight (kg) by height squared (m^2^). Anthropometric measurements were taken in a standing position after subjects removed their heavy clothes. WHR was measured twice, and the mean value was used for analysis. Waist and hip circumferences were measured on bare skin at the level of the umbilicus and iliac crest, respectively, during mid-respiration to the nearest 0.5 cm. The WHR was defined according to the average of two duplicate measurements.

Charts were reviewed to verify other relevant medical conditions, including micro and macrovascular complications of diabetes, and the medication history. A simple satisfaction questionnaire comprising four questions (Do you fell better with metforminXR? Do you think that diarrhea, nausea an vomiting are limiting symptoms to use Metformin ? Do you think that compliance is better using Metformin XR ? Do you want to get back to Metformin?) was applied to the patients at the end of the study.

### Statistical Analysis

The Student t- test was used for comparisons of continuous dependent (intra-group) and continuous independent variables (inter-group). Chi-Square with Yates correction and Fisher's exact test were used for comparison of categorical variables. These analyses were performed using the statistical package for the social sciences (SPSS, version 13.0). Values were expressed as mean (± SD) or median (minimum/maximum). A two-sided P value of less than 0.05 was considered to be significant.

## Results

A total of 177 patients were interviewed for possible enrolment of which 61 patients met the entry criteria. There was a preponderance of women (n = 40) and the age was 54.1 ± 12.1 years with a duration of diabetes of 7.3 ± 6.5 years. Some 28 patients at baseline (45.9%) had gastrointestinal symptoms with persistent use of standard metformin, and diarrhoea was described as the major gastrointestinal side effect in 16 cases. Other patients reported nausea (4 cases), diarrhoea and nausea (4 cases) and epigastric and abdominal pain in a further 2 cases for each. In a quarter of patients the symptoms had been severe enough in the past to precipitate temporary discontinuation of metformin. The majority, 35 patients (54.1%), reported the presence of ghost tablets of metformin XR in their feces during the use of the medication. Microalbuminuria was found in 38 patients (62.2%).

**Figure 1:**
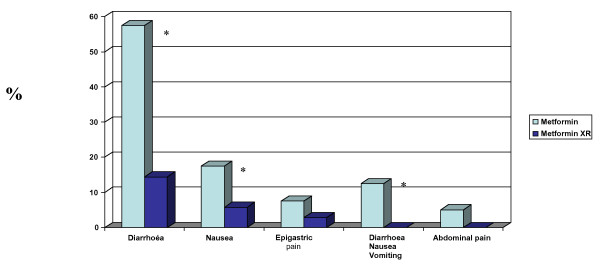
**Incidence of Gastrointestinal side effects before and after switchover to extended release metformin XR in patients completing the 6 month study**. * vs metformin p = 0.013.

Over the 6 months of follow up, a total of 26 patients withdrew from the study including 23 patients who withdrew voluntarily, 2 patients who started on insulin, and 1 patient who unilaterally decided to resume treatment with standard metformin without seeking medical advice. For the remaining group comprising 35 patients, no significant differences were observed for any of the anthropometric, clinical or laboratory measures with the exception of plasma triglycerides, (140.2 +/- 74.8 vs 202.9 +/- 102.2 p = 0.01) following switchover to extended release metformin at 3 and 6 months respectively (Table 1). The metformin daily dose at baseline and following titration of metformin XR was 1861 +/- 711 mg and 1500 +/- 402 mg per day (p = 0.004) respectively. On completion of six months on metformin XR, a total of 27 patients (77.1%) were asymptomatic, with the remainder reporting symptoms of diarrhoea (n = 5), nausea (n = 2) and epigastric pain (n = 1) [Figure 1].

**Table 1:** Clinical and laboratory data at baseline and 3 and 6 months of follow-up

Variables	Baseline	Follow up (90 days)	Follow up (180 days)	p value
	80.78 ± 15.91	79.98 ± 14.98	80.52 ± 15.63	0.12

BMI (kg/m^2^)	31.18 ± 5.42	30.85 ± 5.09	31.03 ± 5.17	0.82

WHR (cm)	101.77 ± 11.24	100.67 ± 10.21	100.53 ± 10.71	0.12

HbA1c (%)	7.61 ± 1.53	7.58 ± 1.44	7.68 ± 1.50	0.713

Cholesterol (mg/dl)	189.33 ± 40.78	191.17 ± 38.39	190.03 ± 42.12	0.945

Triglycerides (mg/dl)	142.53 ± 75.12	186.17 ± 162.79	185.28 ± 139.27	0.088

LDL Chol (mg/dl)	114.59 ± 32.93	109.50 ± 31.18	109.19 ± 33.78	0.463

HDL Chol (mg/dl)	48.51 ± 10.39	48.83 ± 9.07	46.89 ± 10.24	0.242

Vitamin B12 (pg/dl)	293.91 ± 126.54	287.46 ± 122.65	291.57 ± 132.26	0.844

CRP (mg/dl)	0.352 ± 0.31	0.431 ± 0.28	0.372 ± 0.35	0.327

AER (mg/min)	21.04 ± 53.24	27.44 ± 77.76	27.36 ± 75.76	0.309

Data are presented as means ± standard deviation.

Abbreviations: BMI: Body mass index; WHR: Waist/hip ratio;

HbA1C: glycated haemoglobin; CRP: C-reactive protein; AER: Albumin Excretion rate

The results of the patient satisfaction questionnaire indicated that 29 patients (82.9%) felt better on metformin XR whilst one patient reported feeling worse and a further 5 patients stated no treatment preference.

## Discussion

Metformin is recommended as a core therapy in diabetes management worldwide at diagnosis and is seen as being complementary to lifestyle change either alone or in combination with other oral antidiabetic therapies or insulin [8]. The ADA/EASD guidelines mention its use in patients irrespective of age, body weight and degree of baseline hyperglycaemia. Its not therefore surprising that in populations as diverse as this that the use of standard immediate release metformin is problematic in some patient groups leading to difficulties with dose optimization [18]. Limitations to its introduction include multiple dosing and gastrointestinal upsets including diarrhoea, nausea and abdominal pain. A majority of patients treated with metformin are GI symptom free but upwards of 25% of patients experience dose limiting side effects though discontinuation can be in the order of 5%[14]. Gastrointestinal upset can be of a temporary nature but in some cases is low grade and persistent [19]. In the present study, over 77% of patients were GI symptom free on completion of 6 months continuous therapy on extended release metformin. This is noteworthy as almost half the patients at enrolment had persistent GI side effects on standard metformin. Other studies which have examined tolerability outcomes following switchover have reported similar results [20,21]. The extended release metformin tablet releases metformin from the upper GI tract for a longer period of time thereby delaying its entry into the systemic circulation [22,23]. Metered release of metformin from the tablet and administration with the evening meal when gastric residence time is at its longest are thought to be factors in explaining better tolerability.

Switchover to Metformin XR led to a greater sense of well being and patient satisfaction. These findings have the potential to lead to better adherence with therapy. Donnelly and colleagues from Dundee have reported greater adherence in patients switched from standard metformin to an extended release [24]. Moreover, in the same study glycaemic control was reported to improve by almost 1% but small numbers prevented the change becoming significant. According to projections from UKPDS, a 1% decrement in HbA1c is associated with a 14% reduction in risk of heart attacks and a 37% reduction in risk of microvascular complications [25].

We have shown that transfer from standard metformin to the extended release metformin XR tablet is associated with a neutral outcome on cardiovascular risk factors. No detrimental changes were reported for measures of glycaemia, blood pressure. body weight or anthopometric indices and this has been confirmed by others [26].

The same was true for cholesterol, LDL cholesterol and HDL cholesterol. A modest rise was reported for plasma triglycerides and this has been reported before [26,27]. This could reflect the time of administration of metformin XR rather than the formulation. With an evening administration, metformin plasma and tissue concentrations are likely to be higher during the night-time period whereas metformin dosing during the day will lead to higher concentrations at the time of meals and nutrient intake. Plasma triglycerides have been shown to be related to plasma concentrations of metformin [28].

Some concern was expressed by patients that the metformin XR tablet appears to be eliminated in the faeces and is recognizable as a tablet shape. Patients need to be forewarned that this is expected and is a function of the tablet formulation [22]. The extended release properties of the tablet are due to the presence of two insoluble polymers one of which contains metformin. As the tablet passes through the gastrointestinal tract, the tablet swells due to hydration of the outer polymer thereby creating a gel covering. For absorption to take place, metformin must penetrate the outer layer. The intactness of the tablet shape underscores the performance of the extended release tablet.

Switchover to metformin XR had no effect on hs C-reactive protein, a biomarker of chronic inflammatory disease. Previous studies have shown mixed results of metformin therapy on C-reactive protein and overall, studies in non-diabetics [29,30] rather than diabetics [31] have reported greater inhibitory effects. As patients in this study were pre-treated with metformin all that can be concluded is that switchover did not lead to any change. Likewise, no change was reported for vitamin B12 levels following switchover indicating lack of any specific effect of the metformin XR tablet on absorption of this vitamin. Greater understanding of metformin and its interaction with vitamin B12 levels has come from recent studies [32].

Finally, limitations of our study include the open label design with no comparator. However, much is known about metformin having been used in clinical medicine for over 50 years [10]. Of some concern was the large number of patients who left the study, during follow-up but the reasons were mostly social and economic ones arising from repeated attendance at the clinic. Measures of tolerability and patient satisfaction were recorded but using simple questionning techniques rather than validated quality of life measures.

## Conclusions

Switchover to metformin XR resulted in a significant improvement in gastrointestinal tolerability with marked reductions in diarrhoea and nausea. Improved patient satisfaction and well being led to the XR once-a-day tablet being preferred in a high majority of patients. When considered alongside the comparative efficacy of the metformin XR tablet versus standard metformin, this new formulation of metformin has the potential to improve compliance and long-term health outcomes. This is the goal if the aim of therapy is to reduce excess morbidity and mortality in patients with type 2 diabetes.

## Key messages

• Optimising metformin therapy is hindered by multiple daily dosing and persistent gastrointestinal side effects in some patients.

• Extended release metformin is associated with better tolerability and is preferred by patients when given the choice.

• Better compliance will lead to fewer long-term complications and better health outcomes.

## Competing interests

This study have received funding (metformin XR tablets) from Merck Serono (Brazil).

## Authors' contributions

J.L has collected the data; R.A.C has reviwed the data and M.B.G has written the manuscript. All the authors read and approved the final manuscript.
