# Correction: Assessment of efficacy and tolerability of once-daily extended release metformin in patients with type 2 diabetes mellitus

**DOI:** 10.1186/1758-5996-2-57

**Published:** 2010-08-25

**Authors:** Juliana Levy, Roberta A Cobas, Marília B Gomes

**Affiliations:** 1 Department of Medicine, Diabetes Unit, State University of Rio de Janeiro, Rio de Janeiro, Brazil

## Correction

Since publication of our article [1], we have noticed that amendments to the second sentence of the "Study Design" section are required as this should read as follows:

"Approved by the local ethics committee, patient enrolment was restricted to the following patients: those aged 18 years and greater and taking immediate release metformin alone or in combination with other oral agents, those excluded had symptoms of poor diabetes control, were pregnant, had serum creatinine levels >1.3 mg/dL (female) or 1.4 mg/dL (male), nephropathy, evidence of hepatic disease or history of alcohol abuse."

We apologize for any inconvenience or confusion that this may have caused.
